# Induction of haem oxygenase-1 by nitric oxide and ischaemia in experimental solid tumours and implications for tumour growth

**DOI:** 10.1038/sj.bjc.6690624

**Published:** 1999-08

**Authors:** K Doi, T Akaike, S Fujii, S Tanaka, N Ikebe, T Beppu, S Shibahara, M Ogawa, H Maeda

**Affiliations:** Department of Microbiology and 2nd Department of Surgery, Kumamoto University School of Medicine, Kumamoto, 860-0811, Japan; Department of Applied Physiology and Molecular Biology, Tohoku University School of Medicine, Sendai, 980-8575, Japan

**Keywords:** nitric oxide, NO, NO synthase, haem oxygenase-1, tumour growth, vascular permeability, tumour blood flow

## Abstract

Induction of haem oxygenase-1 (HO-1) as well as nitric oxide (NO) biosynthesis during tumour growth was investigated in an experimental solid tumour model (AH136B hepatoma) in rats. An immunohistochemical study showed that the inducible isoform of NO synthase (iNOS) was localized in monocyte-derived macrophages, which infiltrated interstitial spaces of solid tumour, but not in the tumour cells. Excessive production of NO in the tumour tissue was unequivocally verified by electron spin resonance spectroscopy. Tumour growth was moderately suppressed by treatment with either *N*^ω^-nitro-L-arginine methyl ester (L-NAME) or *S*-methylisothiourea sulphate (SMT). In contrast, HO-1 was found only in tumour cells, not in macrophages, by in situ hybridization for HO-1 mRNA. HO-1 expression in AH136B cells in culture was strongly enhanced by an NO (NO^+^) donor *S*-nitroso-*N*-acetyl penicillamine. HO-1 mRNA expression in the solid tumour in vivo decreased significantly after treatment with low doses of NOS inhibitors such as L-NAME and SMT (6–20 mg kg^−1^). However, the level of HO-1 mRNA in the solid tumour treated with higher doses of NOS inhibitor was similar to that of the solid tumour without NOS inhibitor treatment. Strong induction of HO-1 was also observed in solid tumours after occlusion or embolization of the tumour-feeding artery, indicating that ischaemic stress which may involve oxidative stress triggers HO-1 induction in the solid tumour. Lastly, it is of great importance that an HO inhibitor, zinc protoporphyrin IX injected intra-arterially to the solid tumour suppressed the tumour growth to a great extent. In conclusion, HO-1 expression in the solid tumour may confer resistance of tumour cells to hypoxic stress as well as to NO-mediated cytotoxicity. © 1999 Cancer Research Campaign


*British Journal of Cancer* (1999) **80**(12), 1945–1954
© 1999 Cancer Research Campaign
Article no. bjoc.1999.0624

### Induction of haem oxygenase-1 by nitric oxide and ischaemia in experimental solid tumours and implications for tumour growth

**K Doi[1,2], T Akaike[1], S Fujii[1], S Tanaka[1,2], N Ikebe[1,2], T Beppu[2], S Shibahara[3], M Ogawa[2] and H Maeda[1]**

[1]Department of Microbiology and [2]2nd Department of Surgery, Kumamoto University School of Medicine, Kumamoto 860-0811, Japan; [3]Department of Applied Physiology and Molecular Biology, Tohoku University School of Medicine, Sendai 980-8575, Japan

**Summary** Induction of haem oxygenase-1 (HO-1) as well as nitric oxide (NO) biosynthesis during tumour growth was investigated in an experimental solid tumour model (AH136B hepatoma) in rats. An immunohistochemical study showed that the inducible isoform of NO synthase (iNOS) was localized in monocyte-derived macrophages, which infiltrated interstitial spaces of solid tumour, but not in the tumour cells. Excessive production of NO in the tumour tissue was unequivocally verified by electron spin resonance spectroscopy. Tumour growth was moderately suppressed by treatment with either $N^{\omega}$-nitro-L-arginine methyl ester (L-NAME) or *S*-methylisothiourea sulphate (SMT). In contrast, HO-1 was found only in tumour cells, not in macrophages, by in situ hybridization for HO-1 mRNA. HO-1 expression in AH136B cells in culture was strongly enhanced by an NO ($NO^+$) donor *S*-nitroso-*N*-acetyl penicillamine. HO-1 mRNA expression in the solid tumour in vivo decreased significantly after treatment with low doses of NOS inhibitors such as L-NAME and SMT (6–20 mg $kg^{-1}$). However, the level of HO-1 mRNA in the solid tumour treated with higher doses of NOS inhibitor was similar to that of the solid tumour without NOS inhibitor treatment. Strong induction of HO-1 was also observed in solid tumours after occlusion or embolization of the tumour-feeding artery, indicating that ischaemic stress which may involve oxidative stress triggers HO-1 induction in the solid tumour. Lastly, it is of great importance that an HO inhibitor, zinc protoporphyrin IX injected intra-arterially to the solid tumour suppressed the tumour growth to a great extent. In conclusion, HO-1 expression in the solid tumour may confer resistance of tumour cells to hypoxic stress as well as to NO-mediated cytotoxicity.

**Keywords**: nitric oxide; NO; NO synthase; haem oxygenase-1; tumour growth; vascular permeability; tumour blood flow

Expression of different isoforms of nitric oxide (NO)[3] synthase (NOS) has been documented in various tumour cell lines and solid tumours (Buttery et al, 1993; Bastian et al, 1994; Thomsen et al, 1994; Cobbs et al, 1995). Our previous experiments indicate that enhanced vascular permeability in solid tumour is mediated by at least three factors: NO, bradykinin and prostaglandins (Matsumura et al, 1988; Wu et al, 1998; Maeda et al, 1994). NO production by experimental tumours in mice has been implicated in maintenance of tumour blood flow in the neovasculature, and blockade of NO with $N^{\omega}$-nitro-L-arginine has been shown to reduce blood flow in the tumour-associated neovasculature (Andrade et al, 1992; Tozer et al, 1995, 1997).

Among three types of NOS, the inducible isoform (iNOS) produces a much larger amount of NO for a longer time than do the other two constitutive enzymes, i.e. neuronal NOS and endothelial NOS (Stuehr and Griffith, 1992; Moncada and Higgs, 1993). We recently suggested that NO produced in excess in the local area of the solid tumour seems to sustain rapid tumour growth in an experimental solid tumour (Doi et al, 1996). It has been reported, however, that a particularly high output of NO from iNOS is potentially cytotoxic for various tumour cells (Hibbs et al, 1988; Bastian et al, 1994; Lepoivre et al, 1994). The biological effect of NO in tumour biology is thus still unsettled because of previous inconsistent results, i.e. suppressing effects and promoting effects on tumour growth. These contradictory effects of NO may be explained by a putative protective mechanism in the tumour cells against cytotoxicity of excess NO.

In this respect, of considerable interest is a unique biological function of haem oxygenase (HO), originally involved in disintegration of haem compounds in iron metabolism (Tenhunen et al, 1968). HO degrades haem to release carbon monoxide (CO), iron and biliverdin (Maines, 1997). Two isoforms of HO, i.e. HO-1 and HO-2, are known to exist in eukaryotic systems (Maines and Kappas, 1974). HO-1 is induced by various stimuli, such as pro-inflammatory cytokines and heavy metals (Shibahara et al, 1985; Maines, 1997; Yet et al, 1997). Induction of HO-1 has been suggested to provide an important protective response of cells against oxidative damage, because HO-1 induction may decrease the cellular haem level (pro-oxidant) and elevate the level of bilirubin (antioxidant), which is derived from biliverdin and shows a potent scavenging action against reactive oxygen species (Maines, 1997; Kim et al, 1995*a*, 1995*b*). In addition, it is proposed that CO, a product of HO, functions as a gaseous signal transduction molecule in a similar manner to that of NO (Suematsu et al, 1995; Maines, 1997; Prabhakar et al, 1997). It is of interest that HO has been implicated in tumour growth (Lee and Ho, 1994; Hara et al, 1996; Goldman et al, 1996; Takahashi et al, 1996), although details of this mechanism remain unclear.

*Received 30 June 1998*
*Revised 20 January 1999*
*Accepted 28 January 1999*

*Correspondence to:* H Maeda

In the present study, to obtain a better understanding of the role of HO and NO biosynthesis in tumour biology and cancer treatment, we examined the mechanism of induction of HO-1 and iNOS in rat AH136B solid tumours.

### MATERIALS AND METHODS

### Animals and implantation of AH136B tumour

Donryu rats, weighing from 160 to 180 g, were obtained from a commercial supplier (SLC, Inc., Shizuoka, Japan). They were handled according to the guidelines of the Experimental Animal Center of Kumamoto University.

AH136B tumour (hepatoma) cells were cultured serially in ascitic fluid in rats as described previously (Doi et al, 1996). The cells were implanted subcutaneously (s.c.) in a dorsal site of the foot of Donryu rats with an inoculum size of $1 \times 10^7$ cells per injection site. Tumours were allowed to grow and usually reached 15–20 mm in diameter for 16 days.

### Electron spin resonance spectroscopy for analysis of NO production in AH136B solid tumours

Overproduction of NO was examined with the solid tumour obtained 16 days after the tumour cell inoculation by using electron spin resonance (ESR) spectroscopy according to a previously described method (Doi et al, 1996; Setoguchi et al, 1996; Yoshimura et al, 1996). Briefly, a complex of *N*-(dithiocarboxy)-sarcosine (DTCS, 180 mg $kg^{-1}$; Dojindo Laboratories, Kumamoto, Japan) with $FeSO_4.7H_2O$ (40 mg $kg^{-1}$), which is soluble in water, was injected s.c. ESR was performed 30 min after the administration of the iron complex, with the tumour obtained as described below.

The solid tumours obtained with the rats under sodium pentobarbital anaesthesia were transferred to quartz sample tubes (5 mm in diameter). The samples were immediately frozen in liquid nitrogen and then subjected to ESR by using an X-band ESR spectrometer (Bruker 380E) at 110K. Unless otherwise specified, the conditions for ESR measurement were microwave frequency, 9.39 GHz; microwave power, 4 mW; modulation frequency, 100 kHz; and modulation amplitude, 0.5 mT. The magnetic field was calibrated by using TCNQ-Li salt ($g$ = 2.00252).

Simultaneously, to test the effect of NOS inhibitors on the formation of NO in the solid tumour, ESR was performed by using a DTCS-$Fe^{2+}$ complex 1 h after intraperitoneal (i.p.) injection of $N^{\omega}$-nitro-L-arginine methyl ester (L-NAME; Sigma Chemical, St Louis, MO, USA) at 50 mg $kg^{-1}$ or *S*-methylisothiourea sulphate (SMT; Wako Pure Chemical Co., Ltd, Osaka, Japan) at 50 mg $kg^{-1}$.

### Expression and localization of iNOS in AH136B solid tumours

We previously showed that iNOS mRNA expression is highly elevated in AH136B solid tumours implanted and grown in rats (Doi et al, 1996). The localization of iNOS expression was further examined by an immunohistochemical study using a specific antibody for rat iNOS according to our previous method (Setoguchi et al, 1996). Briefly, solid tumour tissue was excised on day 16 after tumour implantation and fixed with 2% periodate–lysine–paraformaldehyde for 6 h. The tissue was then embedded in tissue-embedding medium and frozen in liquid nitrogen, followed by preparation of 6-μm-thick sections by use of a cryostat. After inhibition of endogenous peroxidase activity, the specimens were incubated with an anti-rat iNOS polyclonal antibody (Santa Cruz Biotechnology, Inc., Santa Cruz, CA, USA) for 1 h at room temperature. After the samples were washed with 0.01 M phosphate-buffered 0.15 M saline (PBS, pH 7.4), they were incubated for 1 h with a sheep antimouse immunoglobulin $[F(ab')_2]$ conjugated with peroxidase diluted 1:100. Tissue-bound peroxidase activity was visualized using 3,3′-diaminobenzidine as a substrate; haematoxylin was used for nuclear staining.

In addition, tissue sections were stained immunohistochemically with a specific antibody against exudate monocyte-derived macrophages (TRPM-3) (Takeya et al, 1987). As the control, sections were incubated with non-immunized mouse serum or PBS instead of primary antibody, after which they were processed in the manner just described.

### Expression and localization of HO-1 in AH136B solid tumours

First, expression of HO-1 mRNA was analysed by Northern blotting. Total RNA was extracted from the tumour tissue obtained at 16 days after tumour inoculation by using the guanidine thiocyanate lysis method with Trizol™ reagent (GibcoBRL, Gaithersburg, MD, USA). Each RNA sample (20 μg) underwent electrophoresis on agarose gel and was transferred to the Hybond™-$N^+$ nylon membrane, followed by hybridization of a DNA probe for rat HO-1 mRNA as described previously (Setoguchi et al, 1996; Akaike et al, 1997). The DNA probe was radiolabelled by the random primer technique using [α-$^{32}$P]-dCTP and the Megaprime labelling system™ (Amersham International plc, Buck, UK). An HO-1 cDNA fragment of 882 bp was used for hybridization as reported previously (Takeda et al, 1994), and a cDNA fragment for glyceraldehyde-3-phosphate dehydrogenase (GAPDH) was used as the control-gene expression (Akaike et al, 1997).

Western blot analysis for HO-1 protein was also performed by using a specific polyclonal anti-rat HO-1 antibody (StressGen Biotechnologies, Victoria, Canada). Tumour tissue obtained 16 days after tumour implantation was homogenized, by use of a Polytron homogenizer (Kinematica GmbH, Luzerne, Switzerland), in PBS (pH 7.4) containing 1 mM ethylenediamine tetraacetic acid (EDTA) and 1 mM phenylmethylsulphonyl fluoride (PMSF).

The microsome fraction was obtained by ultracentrifugation at 105 000 ***g*** for 1 h at 4°C. The microsome preparation was then solubilized by adding sodium dodecyl sulphate (SDS), and each aliquot (10 μl; 30 μg of protein content) of the preparation was treated with the buffer for SDS-polyacrylamide gel electrophoresis (PAGE; 10%) under non-reducing conditions as reported earlier (Okamoto et al, 1997). After electrophoresis, protein was transferred to Immobilon™ polyvinylidene difluoride membranes (Millipore Co., Ltd., Bedford, MA, USA) followed by incubation with an anti-HO-1 antibody. The protein band that reacted immunologically with the antibody was visualized by using the ECL™ system (Amersham), combined with chemiluminescence detection with Kodak XAR-5 film.

To localize HO-1 in solid tumour tissue (on day 16 after tumour inoculation), in situ hybridization was performed with use of a digoxigenin RNA labelling kit (Boehringer Mannheim GmbH, Mannheim, Germany) according to the method described

*© 1999 Cancer Research Campaign*

elsewhere (Setoguchi et al, 1996) with some modifications. The digoxigenin-labelled single-strand RNA probe for HO-1 was prepared with an HO-1 cDNA fragment (as described above) as a template. After 10-μm-thick frozen sections of tumour tissue were prepared, they were fixed for 15 min in 4% paraformaldehyde in 0.1 M phosphate buffer and were rinsed in PBS. The sections were then treated serially with 0.001% proteinase K, 4% paraformaldehyde in PBS, and 0.2 M hydrochloric acid (HCl) and were acetylated with 0.25% acetic anhydride in 0.1 M triethanolamine, followed by dehydration through a graded series of ethanol solutions. The sections were then hybridized with either antisense or sense probes for HO-1 mRNA (10 μg $ml^{-1}$ for each) in 100 μl of hybridization solution containing 50% formamide, 0.6 M sodium chloride, 1 mM EDTA, 10% dextran sulphate, 0.25% SDS, 1 × Denhardt's solution, and yeast tRNA (200 μg $ml^{-1}$) in 20 mM Tris–HCl, pH 8.0 at 45°C for 14 h. After hybridization, the sections were washed with 2 × standard saline citrate (SSC) containing 50% formamide at 45°C for 20 min, digested with RNAase (5 μg $ml^{-1}$) at 37°C for 30 min, and washed again with 2 × SSC and then 0.5 × SSC at 45°C. The hybridized digoxigenin-labelled RNA was detected colourimetrically by using a nucleic acid detection kit (Boehringer Mannheim) with a sheep anti-digoxigenin immunoglobulin $[F(ab')_2]$ conjugated with alkaline phosphatase, and nitroblue tetrazolium salt and 5-bromo-4-chloro-3-indolylphosphate for the colour reaction. The phosphatase reaction was carried out in a humidified chamber at 37°C for 1 h.

### Measurement of HO activity in tumour tissues

The HO activity in the AH136B solid tumour or various organs such as livers and spleens was assayed as described previously (Maines and Kappas, 1978). Specifically, the tumour tissues, which was obtained 16 days after tumour implantation, or the liver and spleen were homogenized by the Polytron homogenizer in ice-cold 50 mM potassium phosphate buffer (pH 7.4) containing 2 mM EDTA, 2 mM PMSF and 10 μg $ml^{-1}$ of leupeptin. After the homogenate was centrifuged at 10 000 ***g*** for 30 min at 4°C, the resultant supernatant was used to prepare the microsome fraction as described above (105 000 ***g*** at 4°C). The microsome fraction was suspended in 0.1 M potassium phosphate buffer (pH 7.4), followed by sonication for 2 s at 4°C and stored at –70°C until use. The reaction mixture for the measurement of the HO activity was composed of 2 mg of microsome protein, cytosol fraction of the rat liver (3 mg protein) as a source of biliverdin reductase, 33 μM haemin and 333 μM NADPH in 3 ml of 50 mM potassium phosphate buffer (pH 7.4). The reaction was terminated by addition of 0.1 ml of 0.01 M HCl 15 min after incubation of the reaction mixture. The bilirubin formed in the reaction was extracted with 1 ml of chloroform, and the bilirubin concentration was determined spectroscopically by measuring the difference in absorbance between 464 nm and 530 nm by using a molar extinction coefficient of 40 $mM^{-1}$ $cm^{-1}$ (Maines and Kappas, 1978).

### Culture of AH136B tumour cells and iNOS and HO-1 induction

AH136B cells obtained from the ascitic form of the tumour were cultured in Dulbecco's modified Eagle's medium (DMEM; Gibco, Grand Island, NY, USA) supplemented with non-essential amino acids (Gibco) and 10% fetal bovine serum in 5% carbon dioxide 95% air at 37°C. Cellular iNOS expression was examined by reverse transcriptase-polymerase chain reaction (RT-PCR) and Southern blot analyses. Briefly, total RNA was extracted from a confluent monolayer of the cells in culture on a 6-well polystyrene plate (Falcon, Becton Dickinson Labware, Lincoln Park, NJ, USA; diameter 3.4 cm), and then 3 μg of the RNA were subjected to RT-PCR and Southern blotting for iNOS mRNA. The reaction conditions and oligonucleotide primer used for RT-PCR and a cDNA probe for iNOS are described elsewhere (Doi et al, 1996).

To see whether NO or a related redox form such as nitrosonium ($NO^+$) induces HO-1 mRNA expression in the tumour cells in vitro, *S*-nitroso-*N*-acetyl penicillamine (SNAP; Dojindo Laboratories) was added at various concentrations to the cells in culture (6-well plate) as just described. After a 5 h incubation period at 37°C in the carbon dioxide incubator, total RNA was extracted and 10 μg RNA was used for Northern blotting for HO-1 mRNA in the same manner as for the solid tumour tissues.

### Effect of NOS inhibition on HO-1 induction and solid tumour growth in vivo

To explore the mechanism of HO-1 induction in AH136B solid tumour, HO-1 mRNA expression with or without NOS inhibitors was examined by Northern and Western blot analyses. Specifically, tumour-bearing rats received either L-NAME or SMT in 0.2 ml saline i.p. at a dose of 3, 10, 30, or 45 mg $kg^{-1}$ body weight two times a day for 5 days, beginning 11 days after tumour implantation. The group given only saline served as a control. Solid tumour tissues were obtained 1 day after the last injection of NOS inhibitors, and total RNA was extracted followed by Northern blotting as described above. The microsome fraction of the tissue was isolated by ultracentrifugation and was subjected to Western blotting after SDS-PAGE as noted earlier.

The effect of NOS inhibitors on tumour growth was tested by treatment of tumour-bearing rats with L-NAME or SMT with the same protocol as that used for examination of HO-1 expression. Briefly, after administration of NOS inhibitors, tumour volume was assessed by measuring both major (a) and minor (b) axes of the solid tumours with use of a vernier micrometer. Tumour volume was estimated by use of the formula $(\pi \times a \times b^2)/6$.

### Effect of ischaemic stress on HO-1 expression in AH136B solid tumours in vivo

HO-1 induction after ischaemic treatment was examined by Northern blotting and by measuring its enzyme activity. On day 16 after tumour implantation, the solid tumours were resected at various time points after initiation of surgical occlusion of the iliac artery, which provides the tumour-feeding artery of the tumour-implanted side. Ischaemic stress was also caused in solid tumours by infusion of Lipiodol™ containing styrene-*co*-maleic acid polymer-conjugated neocarzinostatin (Smancs; 0.1 mg of Smancs in 1.0 ml of Lipiodol per 5 min) via cannula (PE10; Becton Dickinson, Sparks, MD, USA) inserted into the feeding artery (iliac artery) (Yamasaki et al, 1987), and the level of HO-1 mRNA expression was monitored as in the occlusion model.

### Treatment of AH136B solid tumours with an HO inhibitor in vivo

To examine the role of HO-1 in the solid tumour growth, zinc protoporphyrin IX (ZnPP) (Wako Pure Chemical, Osaka, Japan)

© 1999 Cancer Research Campaign

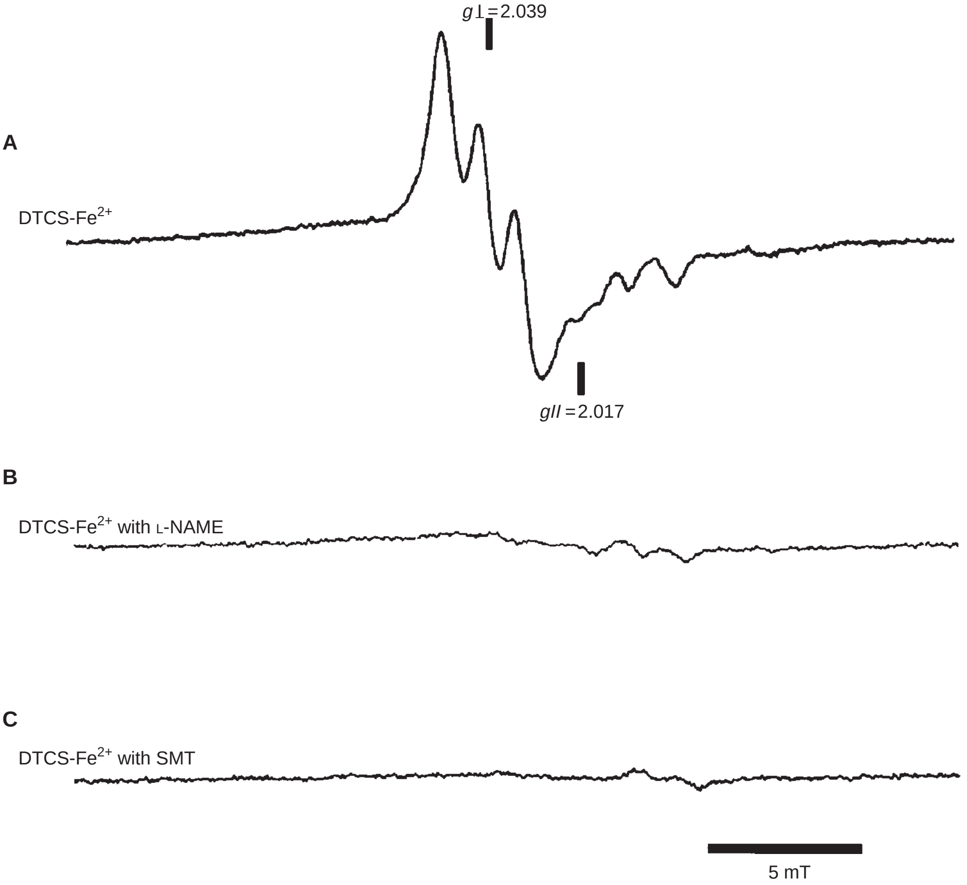

**Figure 1** ESR spectra obtained with AH136B solid tumour tissue. Thirty minutes before the tissue was obtained, a DTCS-$Fe^{2+}$ complex (180–40 mg $kg^{-1}$) was given s.c. to the rats treated (**B**, **C**) or untreated (**A**) with NOS inhibitors (50 mg $kg^{-1}$, i.p.). All ESR measurements were performed at 110 K. See text for details an inhibitor of HO activity (Maines, 1981) was administered to the tumour-bearing animals. Specifically, 0.1 mg of ZnPP in 0.2 ml of 1.0% DMSO in saline was injected intra-arterial to the solid tumour via the feeding artery on day 7 after tumour implantation as just described, and the tumour growth was monitored by measuring the tumour volume in the same manner as the NOS inhibition experiment mentioned above. The group given only 200 µl of 1.0% DMSO in saline served as a control without HO inhibitor treatment.

### Statistical analysis

Data are shown as means ± s.e.m. Statistical difference was analysed by the two-tailed unpaired *t*-test. A *P*-value of < 0.05 was considered statistically significant.

### RESULTS

Excessive production of NO was demonstrated in AH136B solid tumours in rats by ESR spectroscopy: a significant ESR signal of the NO-$(DTCS)_2$-$Fe^{2+}$ adduct was identified (Figure 1). In contrast, only a negligible ESR signal of the NO adduct was observed when animals were given L-NAME or SMT (Figure 1). Because a strong, steady level of induction of iNOS mRNA was observed, we then examined the localization of iNOS in the tumour tissue.

As demonstrated in Figure 2A, an immunohistochemical study revealed that iNOS is expressed only by cells infiltrating the interstitial space, but not by tumour cells. TRPM-3-positive monocyte-derived macrophages were also found to infiltrate the interstitial space of the solid tumour (Figure 2B). The location of iNOS-positive cells appears to be almost the same as that of TRPM-3-positive cells in the tumour tissue. Therefore, iNOS-positive cells were considered to be interstitial infiltrating macrophages rather than AH136B tumour cells. No appreciable immunostaining was observed by the control staining with non-immunized serum or PBS instead of the primary antibodies (anti-iNOS antibody or TRPM-3) (not shown). This result also suggests that monocyte-derived activated macrophages infiltrating in tumour seem to be primarily responsible for overproduction of NO. The lack of iNOS expression in AH136B tumour cells was further substantiated because no appreciable iNOS mRNA expression was observed with AH136B cells in culture by using RT-PCR (Southern blotting) and Northern blotting (data not shown).

Strong HO-1 expression was observed in the solid tumour as assessed by both Northern and Western blot analyses, and by HO activity measurement as well (Figure 3 A–C). HO-1 mRNA expression in the solid tumour in vivo was higher than that in the tumour cells in vitro (Figure 3A). The level of HO-1 induction in the solid tumours was comparable to that in the spleen, which is known to express HO-1 constitutively under physiological conditions (Figure 3 B, C). In contrast to iNOS, HO-1 was observed only in tumour cells of the solid tumour tissue as identified by in situ hybridization with an HO-1 RNA probe (Figure 4).

*© 1999 Cancer Research Campaign*

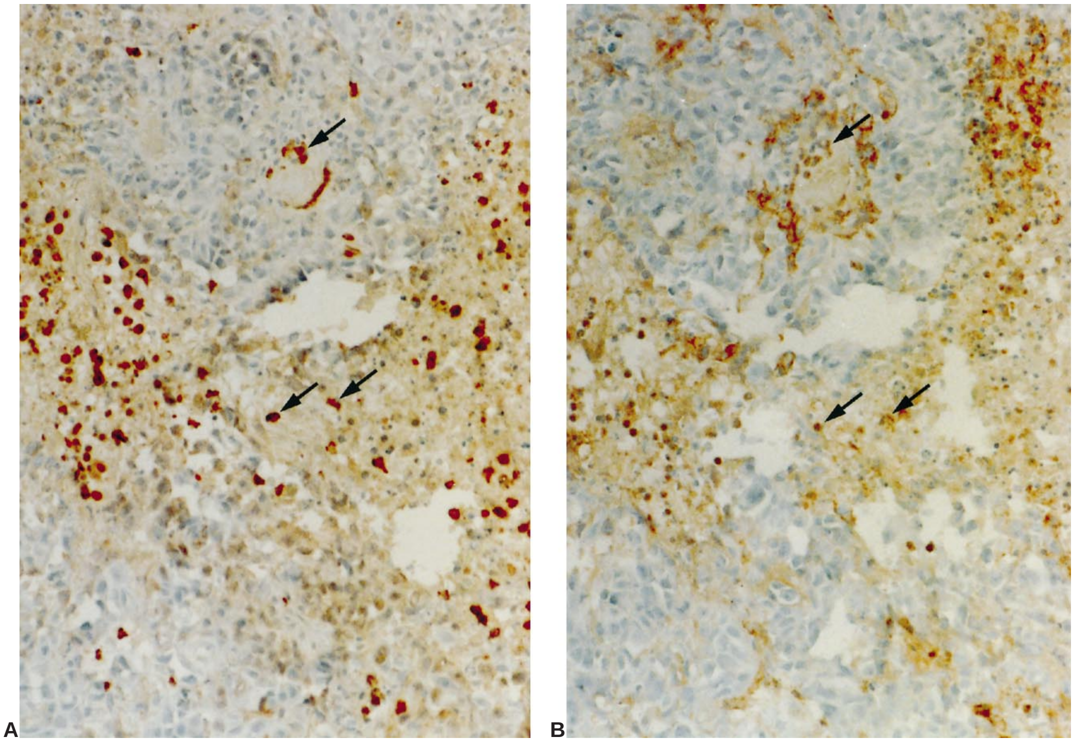

**Figure 2** Immunohistochemistry with antibodies against iNOS and monocyte-derived macrophages (TRPM-3). The localization of iNOS expression was examined in the AH136B solid tumour obtained 16 days after implantation (**A**). With a sequential section of the tumour tissue shown in (**A**), immunostaining was performed with TRPM-3 antibody specific against monocyte-derived exudate macrophages (**B**). Arrows in (**A**) and (**B**) indicate the cells showing apparently consistent localization of the stainings with both anti-iNOS antibody and TRPM-3. Magnification × 170. See text for details

To determine the effect of NO in induction of HO-1, AH136B cells in culture were analysed for expression of HO-1 mRNA after treatment with SNAP in vitro. As shown in Figure 5, induction of HO-1 mRNA was clearly increased in a dose-dependent manner when SNAP was added to the cell culture system. This result indicates that NO or its related redox isoform, $NO^+$, has a potent HO-1-inducing potential in AH136B tumour cells.

The level of HO-1 mRNA in solid tumours in vivo decreased significantly after treatment with low doses (6 and 20 mg $kg^{-1}$) of NOS inhibitors such as L-NAME and SMT. However, as the NOS inhibitor dose increased up to 90 mg $kg^{-1}$ $day^{-1}$, HO-1 mRNA expression gradually recovered to a level similar to that of the solid tumour without treatment (Figure 6). Similar results for the effect of NOS inhibition on HO-1 protein expression were obtained by Western blotting for rat HO-1 (data not shown). The efficacy of NOS inhibition by L-NAME and SMT was tested by an ESR study, and treatment with either L-NAME or SMT abrogated NO production completely in solid tumour tissues as just mentioned (Figure 1 B, C).

To explain the inconsistency in the effect of NO on HO-1 induction in vitro and in vivo, we examined the effect of ischaemic stress on the solid tumours in rats. Ischaemia caused by occlusion of the tumour-feeding artery and by infusion of Smancs/Lipiodol resulted in strong up-regulation of HO-1 mRNA expression (Figure 7A and B). Although there was about a 24-h gap between HO-1 mRNA up-regulation and HO activity induction, the HO activity in the solid tumour was apparently enhanced by the ischaemic stress (Figure 7C).

Moreover, when tumour growth in vivo was investigated with the use of NOS inhibitors, moderate retardation of tumour growth was observed with both L-NAME and SMT (Figure 8), but this effect was not as remarkable as their strong inhibition of NO biosynthesis, as evidenced by ESR spectroscopy (Figure 1 B, C). In contrast, the treatment of HO inhibitor (ZnPP), which significantly inhibited HO activity in the solid tumour, showed a great suppression of the tumour growth in vivo ($P < 0.01$) (Figure 9). In a separate experiment, NO production in the solid tumour with or without ZnPP treatment was investigated by using ESR spectroscopy as just described. ZnPP administered intraarterially, however, did not affect NO biosynthesis by iNOS expressed in the tumour tissue (data not shown).

### DISCUSSION

In our earlier study, excessive production of NO as assessed by ESR spectroscopy correlated well with the rate of growth of AH136B solid tumours in rats (Doi et al, 1996). The present study was performed to further explore this observation and to clarify the role of NO in solid tumour biology, by focusing on the induction and function of HO-1 in AH136B solid tumours.

Rapid tumour growth is sustained by a number of factors derived from tumour cells or a host's tissue in solid tumours (Matsumura et al, 1988; Vaupel et al, 1989; Maeda et al, 1994; Nakano et al, 1996; Suzuki et al, 1996; Wu et al, 1998). Maintenance of regional blood flow in solid tumour tissues appears to be most important for tumour cell growth, for a sufficient supply of molecular oxygen and various nutrients. It is now well known that NO released from vascular endothelial cells plays an important physiological role in regulation of blood flow in systemic circulation via its potent vasodilating action (Moncada

© 1999 Cancer Research Campaign 

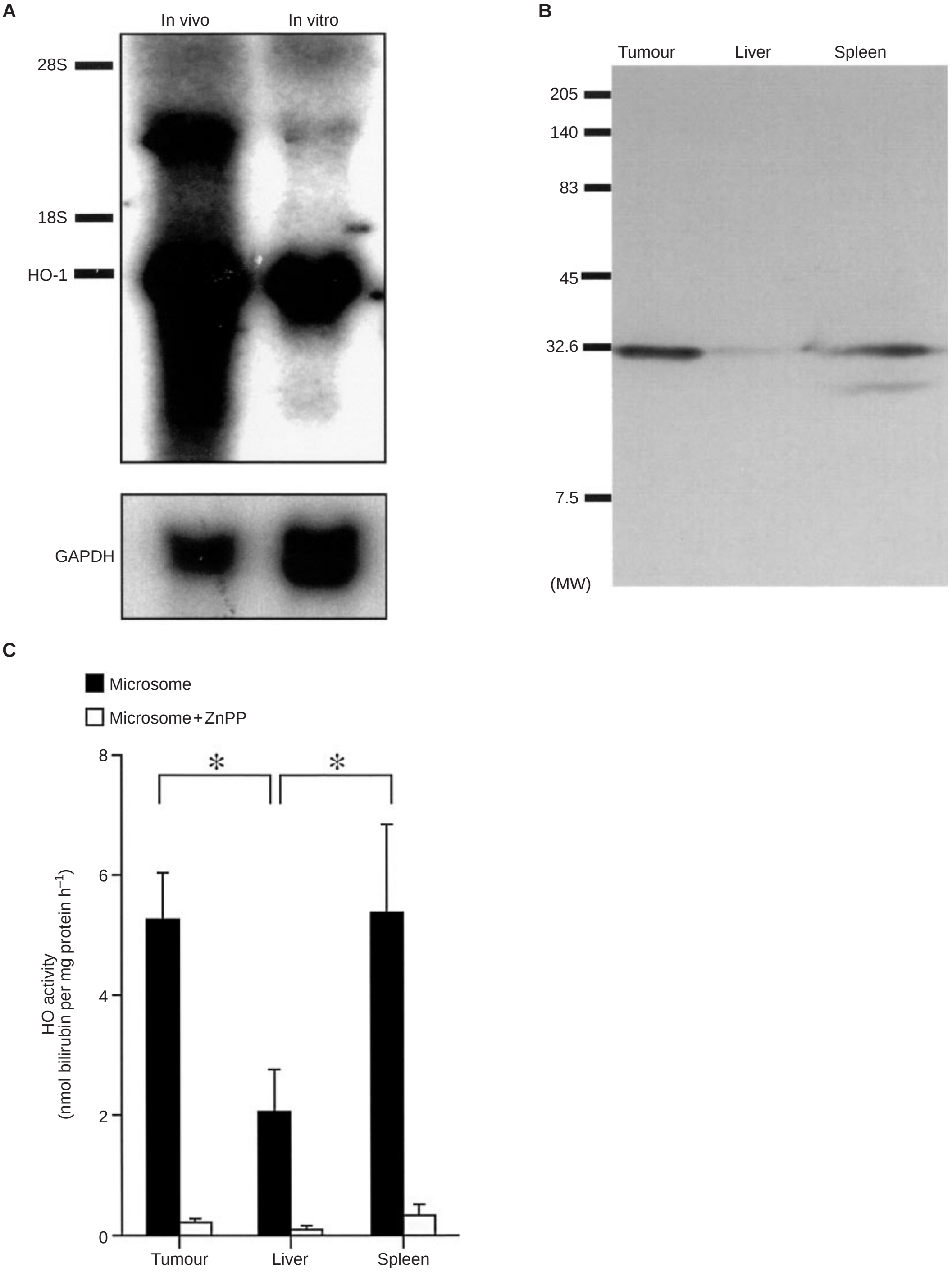

**Figure 3** Northern blot (**A**) and Western blot (**B**) analyses for HO-1 expression and HO activity (**C**) in AH136B tumours. Northern hybridization was performed with AH136B tumours obtained in vitro and in vivo with use of a cDNA probe for rat liver HO-1 (**A**). The expression of glyceraldehyde-3-phosphate dehydrogenase (GAPDH) mRNA in each tissue was determined as for HO-1, as shown in the lower panel. Western blotting was done by using a specific antibody for rat HO-1 with AH136B solid tumour tissue, liver, and spleen (**B**). The HO activity was assayed spectroscopically based on the bilirubin formation from haem in the reaction mixture of the microsome fraction of various tissues in the presence of the rat liver cytosol protein as a source of biliverdin reductase (**C**). Data are shown as means ± s.e.m. ($n = 4$; $*P < 0.05$). Note that the HO activity in each tissue determined was strongly inhibited by addition of 30 μM ZnPP $mg^{-1}$ of microsome protein to the assay mixture. The microsome fraction of the tissue was isolated by ultracentrifugation and was subjected to Western blot analysis after SDS-PAGE and to the measurement for HO activity. See text for details.

and Higgs, 1993). Growth of solid tumour, therefore, may be sustained by increased blood flow mediated by prolonged and excessive production of NO.

Our previous experiment showed that enhanced vascular permeability to tumour tissues was mediated by NO (Maeda et al, 1994; Wu et al, 1998). Leibovich et al (1994) reported that an L-arginine-dependent NO pathway mediates angiogenic activity. Interestingly, Ziche et al (1997) recently reported that vascular endothelial growth factor, VEGF (also called vascular permeability factor, VPF), induces angiogenesis via formation of NO.

*© 1999 Cancer Research Campaign*

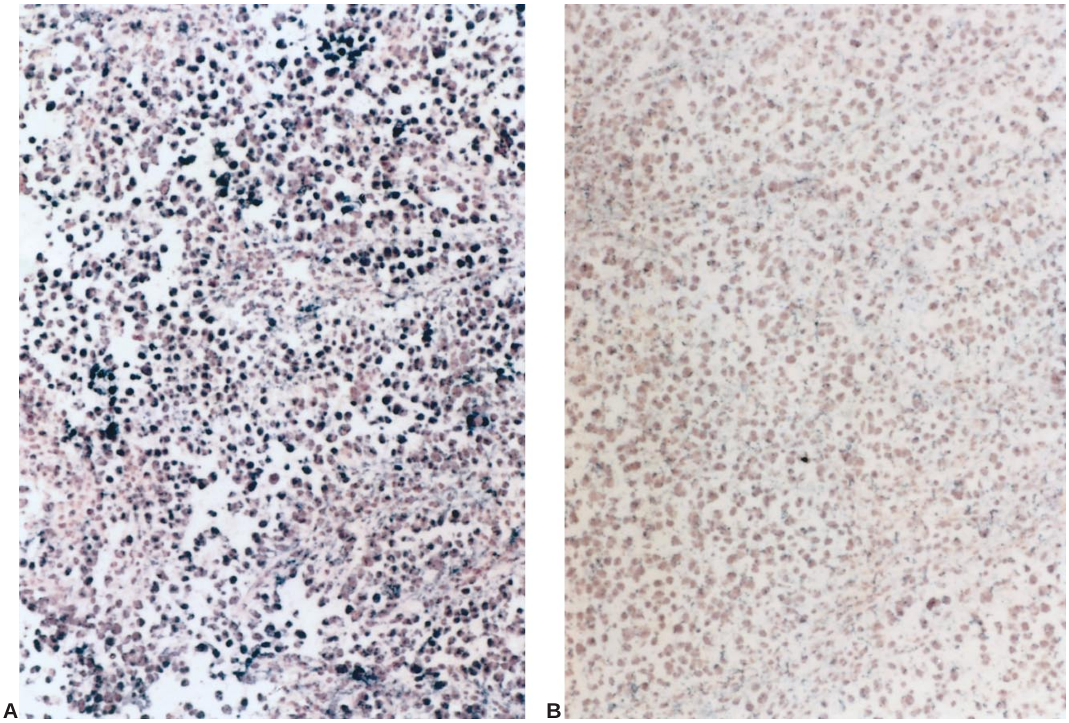

**Figure 4** In situ hybridization for HO-1 expression in AH136B solid tumour. Sections of solid tumour were hybridized with antisense (**A**) or sense (**B**) RNA probes for HO-1 labelled with digoxigenin. AH136B cells are positively stained with the antisense probe for HO-1. Magnification × 130. See text for details

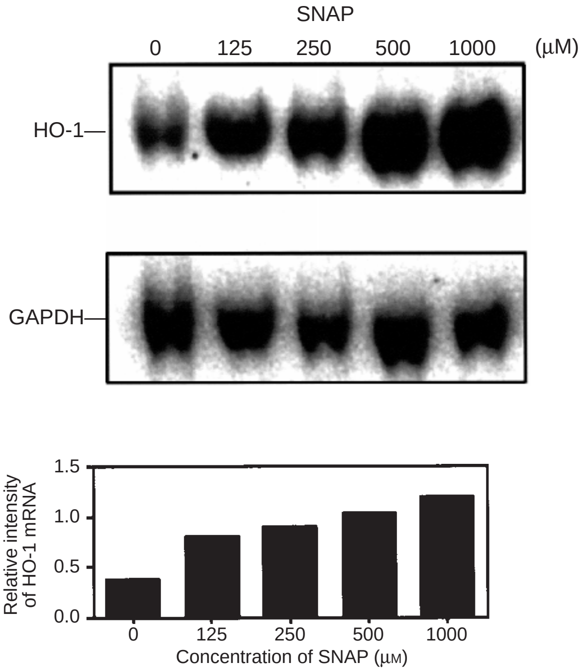

**Figure 5** Northern blot analysis for HO-1 mRNA expression in AH136B tumour cells in culture treated with SNAP. After treatment of cells with various concentrations of SNAP for 5 h, Northern blotting for HO-1 mRNA expression was perfomed in the same manner as described in Figure 3. In the lower panel, the HO-1 mRNA signals were quantified by densitometric analysis, after normalization with GAPDH mRNA signals as a standard mRNA expression, using a Macintosh computer with an Image Scanner (GT6500, Epson Co., Ltd, Tokyo, Japan) and the public domain NIH Image Program. See text for details

Angiogenesis leading to hypervascularization is often observed during rapid tumour growth. Consequently, it is plausible that the angiogenic potential as well as the vascular permeability enhancing effect of NO may facilitate rapid growth of solid tumours, which have great demands for various nutrient factors. A similar beneficial effect of NO on tumour growth was clearly demonstrated recently in tumour-bearing mice, in which human adenocarcinoma cells, which overexpressed iNOS, were implanted (Jenkins et al, 1995). A promoting effect of NO on tumour progression and metastasis is also suggested by Lala and Orucevic (1998). These results are supported by the finding that inhibition of NO synthesis by an NOS inhibitor given systemically reduced tumour growth in mice (Andrade et al, 1992; Thomsen et al, 1997; our present results).

In contrast, it has been reported that NO is an effector molecule in macrophage-mediated cytotoxicity (Hibbs et al, 1988; Bastian et al, 1994; Lepoivre et al, 1994). We showed in the present experiment that the tumour-infiltrating activated macrophage seems to be a major contributor to overproduction of NO in the solid tumour. This observation appears to conflict with the above-mentioned result. To solve these contradictory issues, we probed the mechanism of efficient tumour growth in the presence of an excess amount of NO in the solid tumours.

In this context, Lancaster's group reported that pretreatment of rat hepatocytes with a low dose of NO donor confers resistance to oxidative damage of the cells, through induction of HO-1 (Kim et al, 1995*a*). In their report, it is also demonstrated that HO-1 induction protected the cells from NO-mediated cytotoxicity. HO-1, which catalyses the conversion of haem to biliverdin and CO, has been shown to be constitutively expressed in the liver and spleen and in some tumour cells (Tenhunen et al, 1968; Shibahara et al,

© 1999 Cancer Research Campaign

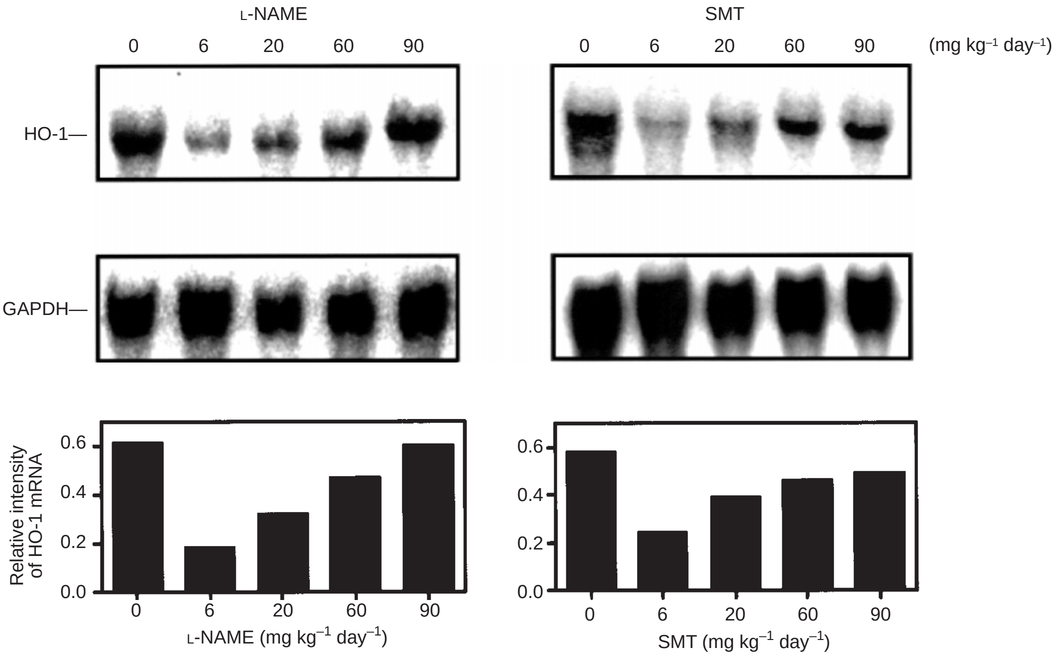

**Figure 6** Northern blot analysis for HO-1 mRNA expression in AH136B solid tumours treated with NOS inhibitors. Tumour-bearing rats received either L-NAME or SMT i.p. at a dose of 3, 10, 30, or 45 mg $kg^{-1}$ two times a day for 5 days, starting 11 days after tumour implantation. Northern blotting for HO-1 mRNA expression was performed in the same manner as described in Figure 3. In the lower panels, quantitative image analysis for HO-1 mRNA was performed in a same manner as in Figure 5. See text for details

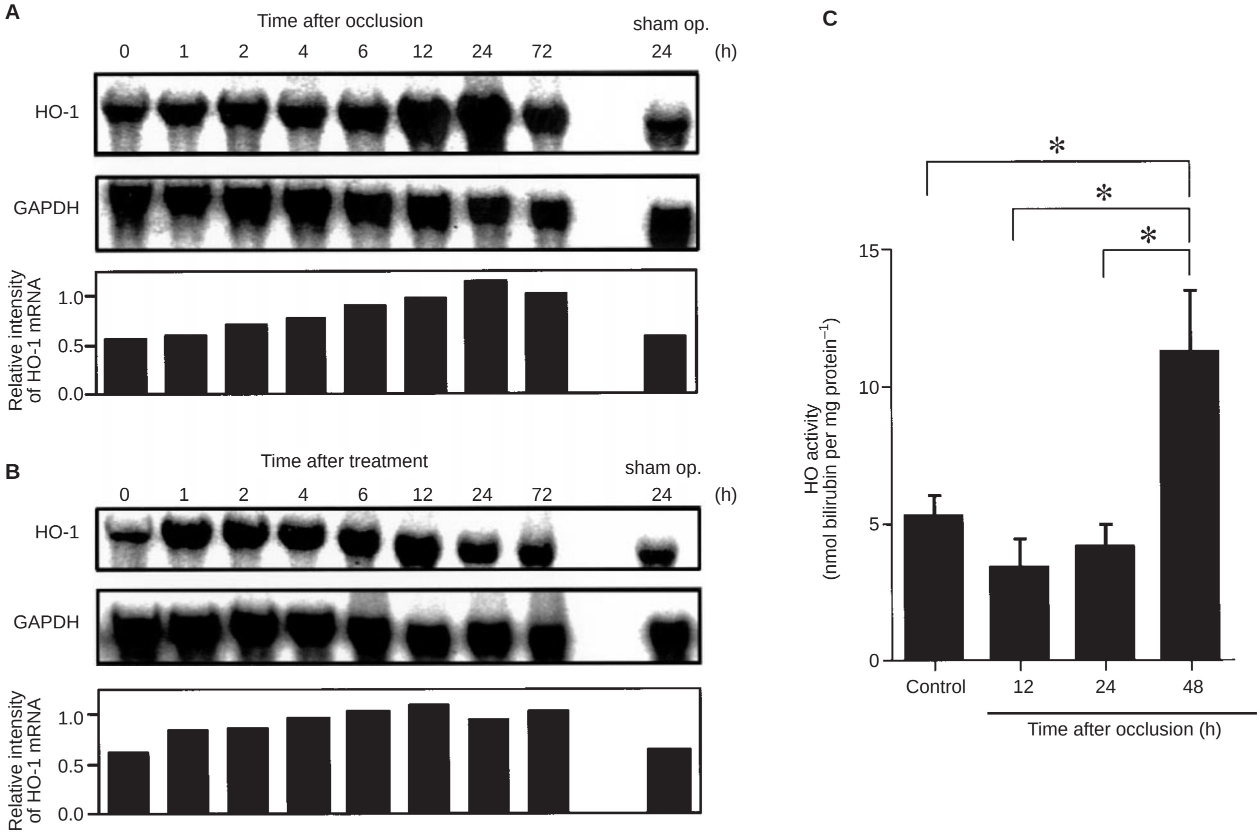

**Figure 7** HO-1 mRNA expression in AH136B solid tumours under ischaemic conditions caused by occlusion of the feeding artery (**A**) or embolization with a Smancs-Lipiodol emulsion (0.1 mg of Smancs in 0.1 ml of Lipiodol) (**B**). Lower panels in (**A**) and (**B**) show the relative signal intensities of HO-1 mRNA quantified similar to Figure 5. (**C**) Induction of HO activity in the solid tumour was also examined after occlusion of the feeding artery in the same manner as in (**A**). Data are expressed means ± s.e.m. ($n = 4$; $*P < 0.05$). Northern blotting was carried out with the solid tumour obtained at various time points after occlusion of the feeding artery or treatment with Smancs-Lipiodol. In a control study (sham operation), HO-1 mRNA expression was examined after laparotomy without occlusion of the feeding artery. See text for details

*© 1999 Cancer Research Campaign*

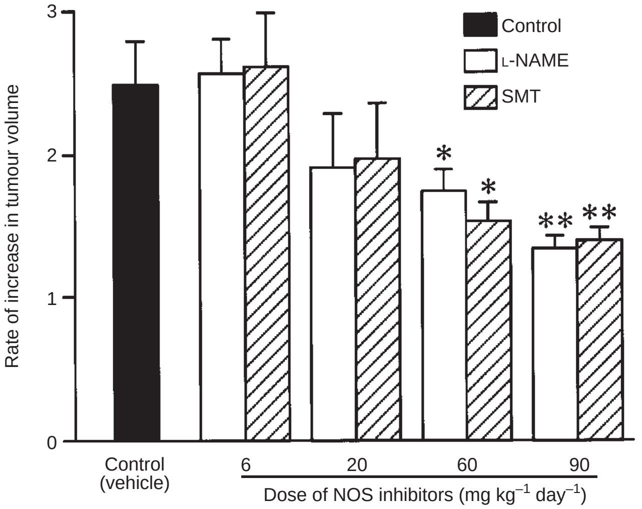

**Figure 8** Effect of HO inhibitor treatment on tumour growth. Tumour-bearing rats received either L-NAME or SMT i.p. at a dose of 3, 10, 30, or 45 mg kg$^{-1}$ two times a day for 5 days, starting 11 days after tumour implantation. The rate of gain in tumour volume was assessed by comparing sizes of both major (a) and minor (b) axes of the solid tumours at 11 and 16 days after tumour cell implantation. The group given only saline served as control. Tumour volumes was estimated by use of the formula $(\pi \times a \times b^2)/6$. Data are shown as means ± s.e.m. (n = 8); asterisks show significant differences versus the control group by the *t*-test for unpaired data (*$P < 0.05$; **$P < 0.01$)

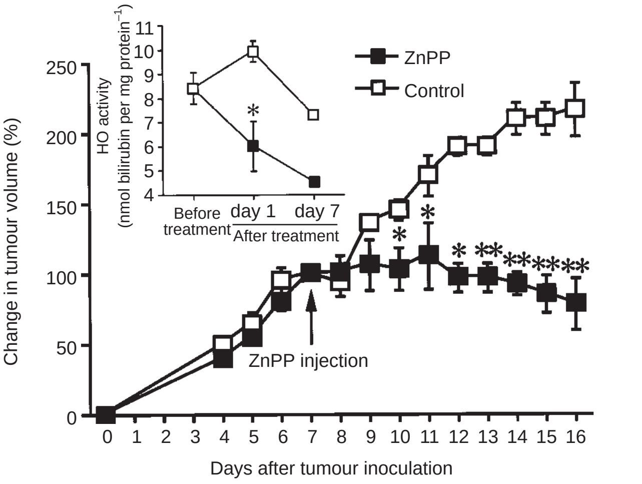

**Figure 9** Effect of HO inhibitor treatment on tumour growth. ZnPP (0.1 mg) in 0.2 ml saline (1.0% DMSO) was administered to the solid tumour via the feeding artery (iliac artery) involving laparotomy at 7 days after tumour implantation. The tumour growth was assessed in the same manner as described in Figure 8. The control group received only 0.2 ml of 1.0% DMSO in saline. Data are shown as means ± s.e.m. ($n = 4$); asterisks show significant differences versus the control group by the *t*-test for unpaired data (*$P < 0.05$; **$P < 0.01$). The inset shows the HO activity in the solid tumour, which was assessed before (7 days after tumour inoculation) and after ZnPP treatment ($n = 4$ except that $n = 1$ for day 7 after ZnPP treatment)

1985; Lee and Ho, 1994; Goldman et al, 1996; Hara et al, 1996; Maines, 1997). Furthermore, HO-1 is readily induced by haem compounds, heavy metals, UV irradiation and a variety of oxidative stresses (Maines and Kappas, 1974; Kim et al, 1995*a*, 1995*b*; Maines, 1997). One possible mechanism of the protective effect of HO-1 induction is thought to be the antioxidant action of bilirubin (Stocker et al, 1987; Kim et al, 1995*a*, 1995*b*), which is generated by reduction of biliverdin, a product of the enzymatic reaction of HO using haem as a substrate. In addition, ferritin, whose expression was triggered by iron release during haem breakdown catalysed by HO-1, has been proposed to protect against oxidative stress by sequestering iron (Kim et al, 1995*b*). On the basis of these findings related to the role of HO-1 induction in cellular defense mechanisms, induction of HO-1 may benefit the tumour cells themselves.

In fact, in our present experiment with a solid tumour model in rats, HO-1 was constantly up-regulated. The level of HO-1 mRNA expression was apparently enhanced in AH136B tumour cells in culture by treatment with an NO ($NO^+$) donor. Regulation of HO-1 expression by NO was also verified in AH136B tumour-bearing rats in vivo, as revealed by suppression of HO-1 induction by NOS inhibitor treatment of the animals. However, reduction in HO-1 expression was only marginal when higher doses (more than 60 mg kg$^{-1}$) of NOS inhibitor were administered, in which NO biosynthesis will be completely inhibited in AH136B solid tumour tissues. This bell shape suppression of HO-1 expression by NOS inhibitors in the solid tumour may be explained by the fact that ischaemic or hypoxic stress is also a potent inducer of HO-1 in AH136B solid tumour in rats, as seen in Figure 7. Specifically, a complete inhibition of NO synthesis by NOS inhibitors led to profound reduction of tumour blood flow and thus resulted in an ischaemic insult to AH136B solid tumours, which may then trigger up-regulation of HO-1 in the tissue even in the absence of stimulation with NO. In this context, it is reported that hypoxia is indeed caused by an NOS inhibitor ($N^{\omega}$-nitro-L-arginine) in experimental murine tumours (Wood et al, 1994).

In addition, we observed only moderate suppression of tumour growth in vivo with NOS inhibitor treatment. In view of our present finding that not only NO but also ischaemic stimuli can be the major inducers of HO-1 expression in solid tumours, it may be that HO-1 has a compensatory function to sustain effective tumour growth even after suppression of the potent vasoactive NO. Moreover, it is of considerable importance that intra-arterial administration of the HO inhibitor ZnPP strongly suppressed the AH136B tumour growth in vivo (Figure 9). This result supports the above notion that compensatory HO-1 induction in the solid tumour may be advantageous for tumour cell growth.

In conclusion, our current study indicates that HO-1 expression in solid tumour appears to be regulated by NO and ischaemic stress, which should often occur in tumour tissue during rapid tumour growth in vivo. The up-regulation of HO-1 may have protective and beneficial effects for tumour cells against anti-tumour actions of the host as well as many anticancer agents.

### ACKNOWLEDGEMENTS

The authors thank Ms Judith Gandy for editing and Ms Rie Yoshimoto for preparing the manuscript. Thanks are due also to Dr Motohiro Takeya, Department of Pathology, Kumamoto University School of Medicine, for his help in the immunohistochemical analysis. This work was supported by a Grant-in-Aid for Research on Cancer from the Ministry of Education, Culture, Sports and Science of Japan.

© 1999 Cancer Research Campaign 

### REFERENCES

Akaike T, Inoue K, Okamoto T, Nishino H, Otagiri M, Fujii S and Maeda H (1997) Nanomolar quantification and identification of various nitrosothiols by high performance liquid chromatography coupled with flow reactors of metals and Griess reagent. *J Biochem* **122**: 459–466

Andrade SP, Hart IR and Piper PJ (1992) Inhibitors of nitric oxide synthase selectively reduce flow in tumor-associated neovasculature. *Br J Pharmacol* **107**: 1092–1095

Bastian NR, Yim CY, Hibbs Jr JB and Samlowski WE (1994) Induction of iron-derived EPR signals in murine cancers by nitric oxide. Evidence for multiple intracellular targets. *J Biol Chem* **269**: 5127–5131

Buttery LDK, Springall DR, Andrade SP, Riveros-Moreno V, Hart I, Piper PJ and Polak JM (1993) Induction of nitric oxide synthase in the neo-vasculature of experimental tumors in mice. *J Pathol* **171**: 311–319

Cobbs CS, Brenman JE, Aldape KD, Bredt DS and Israel MA (1995) Expression of nitric oxide synthase in human central nervous system tumors. *Cancer Res* **55**: 727–730

Doi K, Akaike T, Horie H, Noguchi Y, Fujii S, Beppu T, Ogawa M and Maeda H (1996) Excessive production of nitric oxide in rat solid tumor and its implication in rapid tumor growth. *Cancer* **77**: 1598–1604

Goldman AI, Choudhury M, da Silva JL and Jiang S (1996) Quantitative measurement of haem oxygenase-1 in the human renal adenocarcinoma. *J Cell Biochem* **63**: 342–348

Hara E, Takahashi K, Tominaga T, Kumabe T, Kayama T, Suzuki H, Fujita H, Yoshimoto T, Shirato K and Shibahara S (1996) Expression of haem oxygenase and inducible nitric oxide synthase mRNA in human brain tumors. *Biochem Biophys Res Commun* **224**: 153–158

Hibbs JB, Taintor RR, Varin Z and Rachlin EM (1988) Nitric oxide: a cytotoxic activated macrophage effector molecule. *Biochem Biophys Res Commun* **157**: 87–94

Jenkins DC, Charles IG, Thomsen LL, Moss DW, Holmes LS, Baylis SA, Rhodes P, Westmore K, Emson PC and Moncada S (1995) Role of nitric oxide in tumor growth. *Proc Natl Acad Sci USA* **92**: 4392–4396

Kim Y-M, Bergonia H and Lancaster Jr JR (1995*a*) Nitrogen oxide-induced autoprotection in isolated rat hepatocytes. *FEBS Lett* **374**: 228–232

Kim Y-M, Bergonia HA, Müller C, Pitt BR, Watkins WD and Lancaster Jr JR (1995*b*) Loss and degradation of enzyme-bound haem induced by cellular nitric oxide synthesis. *J Biol Chem* **270**: 5710–5713

Lala PK and Orucevic A (1998) Role of nitric oxide in tumor progression: lessons from experimental tumors. *Cancer Metastasis Rev* **17**: 91–106

Lee TC and Ho IC (1994) Expression of haem oxygenase in arsenic-resistant human lung adenocarcinoma cells. *Cancer Res* **54**: 1660–1664

Leibovich SJ, Polverini PJ, Fong TW, Harlow LA and Koch AE (1994) Production of angiogenic activity by human monocytes requires an L-arginine/nitric oxide-synthase-dependent effector mechanism. *Proc Natl Acad Sci USA* **91**: 4190–4194

Lepoivre M, Flamn JM, Bobe P, Lemaire G and Henry Y (1994) Quenching of the tyrosyl free radical of ribonucleotide reductase by nitric oxide. Relationship to cytostasis induced in tumor cells by cytotoxic macrophage. *J Biol Chem* **269**: 21891–21897

Maeda H, Noguchi Y, Sato K and Akaike T (1994) Enhanced vascular permeability in solid tumor is mediated by nitric oxide and inhibited by both new nitric oxide scavenger and nitric oxide synthase inhibitor. *Jpn J Cancer Res* **85**: 331–334

Maines MD (1981) Zinc-protoporphyrin is a selective inhibitor of haem oxygenase activity in the neonatal rat. *Biochim Biophys Acta* **673**: 339–350

Maines MD (1997) The haem oxygenase system: a regulator of second messenger gases. *Ann Rev Pharmacol Toxicol* **37**: 517–554

Maines MD and Kappas A (1974) Cobalt induction of hepatic haem oxygenase: with evidence that cytochrome P-450 is not essential for this enzyme activity. *Proc Natl Acad Sci USA* **71**: 4293–4297

Maines MD and Kappas (1978) Prematurely evoked synthesis and induction of δ-aminolevulinate synthetase in neonatal liver. *J Biol Chem* **253**: 2321–2326

Matsumura Y, Kimura M, Yamamoto T and Maeda H (1988) Involvement of the kinin-generating cascade in enhanced vascular permeability in tumor tissue. *Jpn J Cancer Res* **79**: 1327–1334

Moncada S and Higgs A (1993) The L-arginine-nitric oxide pathway. *N Engl J Med* **329**: 2002–2012

Nakano S, Matsukado K and Black KL (1996) Increased brain tumor microvessel permeability after intracarotid bradykinin infusion is mediated by nitric oxide. *Cancer Res* **56**: 4072–4031

Okamoto T, Akaike T, Nagano T, Miyajima S, Suga M, Ando M, Ichimori K and Maeda H (1997) Activation of human neutrophil procollagenase by nitrogen dioxide and peroxynitrite: a novel mechanism for procollagenase activation involving nitric oxide. *Arch Biochem Biophys* **342**: 261–274

Prabhakar NR, Dinerman JL, Agani FH and Snyder SH (1997) Carbon monoxide: a role in carotid body chemoreception. *Proc Natl Acad Sci USA* **92**: 1994–1997

Setoguchi K, Takeya M, Akaike T, Suga M, Hattori R, Maeda H, Ando M and Takahashi K (1996) Expression of inducible nitric oxide synthase and its involvement in pulmonary granulomatous inflammation in rats. *Am J Pathol* **149**: 2005–2022

Shibahara S, Müller R, Taguchi H and Yoshida T (1985) Cloning and expression of cDNA for rat haem oxygenase. *Proc Natl Acad Sci USA* **81**: 7865–7869

Stocker R, Yamamoto Y, McDonagh AF, Glazer AN and Ames BN (1987) Bilirubin is an antioxidant of possible physiological importance. *Science (Wash. DC)* **235**: 1043–1046

Stuehr DJ and Griffith OW (1992) Mammalian nitric oxide synthases. *Adv Enzymol Relat Areas Mol Biol* **65**: 287–346

Suematsu M, Goda N, Sano T, Kashiwagi S, Egawa T, Shinoda Y and Ishimura Y (1995) Carbon monoxide: an endogenous modulator of sinusoidal tone in the perfused rat liver. *J Clin Invest* **96**: 2431–2437

Suzuki K, Hayashi N, Miyamoto M, Yamamoto M, Ohkawa K, Ito Y, Sasaki Y, Yamaguchi Y, Nakase H, Noda K, Enomoto N, Arai K, Yamada Y, Yoshihara H, Tsujimura T, Kawano K, Yoshikawa K and Kamada T (1996) Expression of vascular permeability factor/vascular endothelial growth factor in human hepatocellular carcinoma. *Cancer Res* **56**: 3004–3009

Takahashi K, Hara E, Suzuki H, Sasano H and Shibahara S (1996) Expression of haem oxygenase isozyme mRNAs in the human brain and induction of haem oxygenase-1 by nitric oxide donors. *J Neurochem* **67**: 482–489

Takeda A, Onodera H, Sugimoto A, Itoyama Y, Kogure K and Shibahara S (1994) Increased expression of haem oxygenase mRNA in rat brain following transient forebrain ischemia. *Brain Res* **666**: 120–124

Takeya M, Hsiao L and Takahashi K (1987) A new monoclonal antibody, TRPM-3, binds specifically to certain rat macrophage populations. Immunohistochemical and immunoelectron microscopic analysis. *J Leukoc Biol* **41**: 187–195

Tenhunen R, Marver HS and Schmid R (1968) The enzymatic conversion of haem to bilirubin by microsomal haem oxygenase. *Proc Natl Acad Sci USA* **61**: 748–755

Thomsen LL, Lawton FG, Knowles RG, Beesley JE, Riveros-Moreno V and Moncada S (1994) Nitric oxide synthase activity in human gynecological cancer. *Cancer Res* **54**: 1352–1354

Thomsen LL, Scott JMJ, Topley P, Knowles RG, Keerie A-J and Frend AJ (1997) Selective inhibition of inducible nitric oxide synthase inhibits tumor growth in vivo: studies with 1400W, a novel inhibitor. *Cancer Res* **57**: 3300–3304

Tozer GM, Prise VE and Bell KM (1995) The influence of nitric oxide on tumor vascular tone. *Acta Oncol* **34**: 373–377

Tozer GM, Prise VE and Chaplin DJ (1997) Inhibition of nitric oxide synthase induces a selective reduction in tumor blood flow that is reversible with L-arginine. *Cancer Res* **57**: 948–955

Vaupel P, Kallinowski F and Okunieff P (1989) Blood flow, oxygen and nutrient supply, and metabolic microenvironment of human tumors: a review. *Cancer Res* **49**: 6449–6465

Wood PJ, Sansom JM, Butler SA, Stratford IJ, Cole SM, Szabó C, Thiemermann C and Adams GE (1994) Induction of hypoxia in experimental murine tumors by the nitric oxide synthase inhibitor, $N^G$-nitro-L-arginine. *Cancer Res* **54**: 6458–6463

Wu J, Akaike T and Maeda H (1998) Modulation of enhanced vascular permeability in tumor by a bradykinin antagonist, a cyclooxygenase inhibitor, and an NO scavenger. *Cancer Res* **58**: 159–165

Yamasaki K, Konno T, Miyauchi Y and Maeda H (1987) Reduction of hepatic metastasis in rabbits by administration of an oily anticancer agent into the portal vein. *Cancer Res* **47**: 852–855

Yet S-F, Pellacani A, Patterson C, Tan L, Folta SC, Foster L, Lee W-S, Hsieh C-M and Perella MA (1997) Induction of haem oxygenase-1 expression in vascular smooth muscle cells. A link to endotoxic shock. *J Biol Chem* **272**: 4295–4301

Yoshimura T, Yokoyama H, Fuji S, Takayama F, Oikawa K and Kamada H (1996) In vivo ESR detection and imaging of endogenous nitric oxide in lipopolysaccharide-treated mice. *Nature Biotechnol* **14**: 992–994

Ziche M, Morbidelli L, Choudhuri R, Zhang H-T, Donnini S, Granger HJ and Bicknell R (1997) Nitric oxide synthase lies downstream from vascular endothelial growth factor-induced but not basic fibroblast growth factor-induced angiogenesis. *J Clin Invest* **99**: 2625–2634

 *© 1999 Cancer Research Campaign*
